# Design and Analysis of a Combined Strain–Vibration–Temperature Sensor with Two Fiber Bragg Gratings and a Trapezoidal Beam

**DOI:** 10.3390/s19163571

**Published:** 2019-08-16

**Authors:** Kun Yao, Qijing Lin, Zhuangde Jiang, Na Zhao, Gang-Ding Peng, Bian Tian, Wenyun Jia, Ping Yang

**Affiliations:** 1 State Key Laboratory of Mechanical Manufacturing Systems Engineering, Xi’an Jiaotong University, Xi’an 710049, China; 2 Collaborative Innovation Center of High-End Manufacturing Equipment, Xi’an Jiaotong University, Xi’an 710054, China; 3 State Key Laboratory of Mechanical System and Vibration, Shanghai Jiaotong University, Shanghai 200240, China; 4 Xi’an Jiaotong University Suzhou Institute, Suzhou 215123, China; 5 School of Electrical Engineering and Telecommunications, UNSW, Sydney, NSW 2052, Australia; 6 Northwest A&F University, Xianyang 712100, China

**Keywords:** combined FBG sensor, trapezoidal beam, vibration sensing, strain sensing, temperature sensing, simultaneously sensing

## Abstract

A combined sensor to simultaneously measure strain, vibration, and temperature has been developed. The sensor is composed of two Fiber Bragg gratings (FBGs) and a vibration gainer. One FBG is used to measure strain, while the other measures vibration and temperature. The gainer has a mass block which is used to increase its sensitivity to vibration. The main beam of the vibration gainer was designed as a trapezoid in order to reduce the strain gradient while sensing vibration. In addition, an interrogation method was used to eliminate interactions between measured parameters. Experiments were carried out to analyze the performance of the proposed sensor. For individual strain measurement in the range of 0–152 με, the sensitivity and nonlinearity error were 1.878 pm/με and 2.43% Full Scale (F.S.), respectively. For individual temperature measurement in the range of 50–210 °C, the sensitivity and nonlinearity error were 29.324 pm/°C and 1.88% F.S., respectively. The proposed sensor also demonstrated a sensitivity of 0.769 pm/m·s^−2^ and nonlinearity error of 1.83% F.S. for vibration measurement in the range of 10–55 m/s^2^. Finally, simultaneously measuring strain, temperature, and vibration resulted in nonlinearity errors of 4.23% F.S., 1.89% F.S., and 2.23% F.S., respectively.

## 1. Introduction

Vibration, strain, and temperature are important parameters for assessing the health of mechanical structures [1,2,3]. Different types of sensors have been developed to measure these parameters. Jun et al. [4] developed a strain sensor based on individual ZnO piezoelectric fine-wires for monitoring structures. Moreover, Morteza et al. [5] designed a high sensitivity and stretchable strain sensor based on silver nanowire. Scott et al. [6] developed a MEMS-based temperature sensor to monitor the health of engine components. Basten et al. [7] presented a network of wireless vibration sensors for monitoring the health of structures.

In addition to electronic sensors, optical fiber sensors have been developed [8,9,10,11]. In particular, fiber Bragg grating (FBG) sensors are widely used owing to several advantages such as their small size, insensitivity to electromagnetic interference, resistance to corrosion, and suitability for multi-parameter sensing and multiplexing [12,13,14,15]. However, measured parameters can interact with each other during sensing, therefore, various structures have been proposed to reduce these interactions. For instance, Xu et al. [16] presented a temperature-independent strain sensor with a chirped Bragg grating in a tapered optical fiber and used reflected light intensity to demodulate strain signals. However, accuracy of the sensor is easily affected by bending of the fiber. Khan et al. [17] presented an FBG sensor with self-temperature compensation. The sensor consists of two FBGs and an L-shaped cantilever that can only be used to measure vibration. Mikel et al. [18] presented a strain sensor with two FBGs and an aluminum structure that can also achieve self-temperature compensation. However, the results can be unreliable, since aluminum structure places additional strain on the measured object. Mizutani et al. [19] presented another self-temperature compensated FBG sensor to measure the average strain, strain distribution, and vibrations. However, the sensor cannot simultaneously measure parameters and the range of vibration that can be measured is limited by the scanning frequency of the Fabry-Pérot interferometer, which was set as 100 Hz in the experiment. Salo et al. [20] performed a pre-evaluation of two types of FBG sensors for measuring beam deflection and vibration and found that the sensor must be 100 cm long and 40 mm high to achieve an accuracy of 0.2 mm. Moreover, the reading frequency of 2 Hz is quite low. Jiang et al. [21] proposed a three-component FBG sensor to measure vibration, temperature, and verticality. The frequency measurement error was in the range of 100–1000 Hz, which is less than 1%, and the maximum temperature sensitivity was 13.4 pm/°C in the range of 20–60 °C. However, application of the sensor is somewhat limited, since it needs to be complexly packaged.

In general, existing sensors cannot detect vibration, strain, and temperature simultaneously unless multiple sensors are used. Moreover, some sensors can only achieve temperature compensation by adding an extra temperature sensor, which does not promote sensor integration. In this work, a combined FBG sensor was developed that can measure vibration, strain, and temperature simultaneously and can achieve self-temperature compensation. This reduces the number of used sensors, thereby minimizing the total space occupied by sensors. Moreover, this can simplify the layout of sensors. The developed sensor is composed of two FBGs and a specially designed vibration gainer, which increases the sensitivity of vibration measurement. Finally, interactions between measured parameters are eliminated by using an interrogation method.

## 2. Sensor Design

### 2.1. Sensor Structure

**Figure 1:**
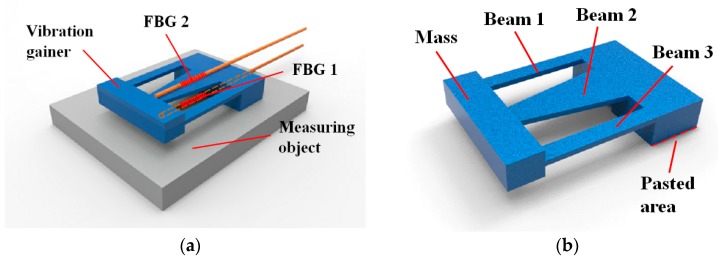
Diagram of sensor. (**a**) Sensor components. (**b**) Structure of vibration gainer.

The designed sensor consists of two FBG sensors, FBG 1 and FBG 2, and a vibration gainer, as shown in Figure 1. The FBG 1 is attached to the surface of a measuring object and used to measure strain. When strain is generated on the surface of the object due to tensile or pressure stress, the FBG 1 is stretched or compressed and its center wavelength changes accordingly [22]. However, a corresponding change in temperature can cause the center wavelength of FBG 1 to change, which can lead to errors in the strain measurement. The vibration gainer is also attached to the measuring object on top of the FBG 1. The gainer is comprised of a cuboid mass and three cantilever beams. The mass is used to increase the sensitivity of vibration sensing. The main beam (beam 2) is designed as a trapezoid to increase the uniformity of strain on the surface [23]. The side beams (beam 1 and beam 3) improve torsion resistance of the structure, thereby helping to avoid torsional deformation.

Owing to vibrations, alternating strains are generated on the upper surface of the beams. The FBG 2 is attached to beam 2 using an adhesive. By monitoring the center wavelength of FBG 2, the frequency and acceleration of vibration can be obtained. Temperature can influence the spectrum of FBG 2 as well. Compared to vibration, temperature changes slowly, thus, it can be considered constant over a short time period and the Fast Fourier Transform (FFT) can be used to separate temperature and vibration. The value at 0 Hz of the frequency spectrum, obtained by FFT, represents the temperature. Values at all other frequencies represent vibrations. However, the value at 0 Hz is usually much larger than the other values, which makes it difficult to detect and distinguish vibration signals.

The interrogation method proposed in this paper isolates a one-second-long signal from the signal sensed by FBG 2 and subtracts its average value before it is analyzed by FFT. Therefore, vibration values can be easily detected and distinguished. The average value represents temperature. After the frequency spectrum is obtained by FFT, the values at non-zero frequencies represent vibrations. Once the temperature is obtained, it can be used to modify the strain measured by FBG 1, which will be discussed further in Section 3.1.2.

The values mentioned above are, in fact, changes of reflection centers of FBGs. Since the sensing system and analyzing system can work simultaneously, this signal separation method can be used for dynamic vibration detection.

### 2.2. Theoretical Analysis of Surface Strain of the Beam

**Figure 2:**
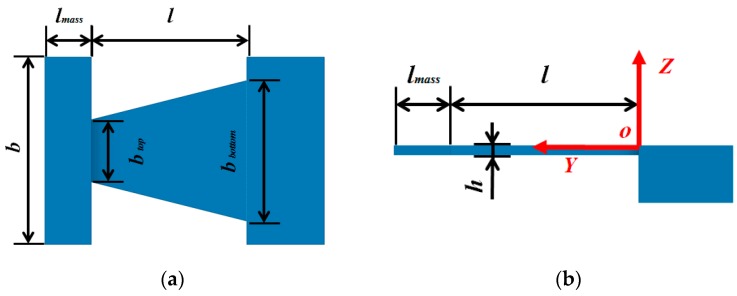
Simplified beam for theoretical analysis. (**a**) Top view. (**b**) Side view.

To simplify the calculation, beam 1, beam 2, and beam 3 are combined to form a wide trapezoidal cantilever beam, as shown in Figure 2. The thickness of the mass is modified to be the same thickness as the beam, and the density of the mass increases in order to maintain its original quality. Then the response of the simplified cantilever beam to arbitrary load can be calculated by the following equation [24]:

(1) $$z(y,t)=\sum_{j=1}^{\infty }\frac{1}{\omega_{j}}Y_{j}(y)\int_{0}^{l+l_{mass}}Y_{j}(y)\times \int_{0}^{t}[p(y,\tau )-\frac{\partial }{\partial y}m(y,\tau )]\sin\omega_{j}(t-\tau )d\tau dy$$

where *z* and *y* are the coordinates; z(y,t) represents the deflection in the *Z* direction; $\omega_{j}$ is the natural frequency of order *j*; $Y_{j}(y)$ is the main vibration type of order *j*; *l* is the length of the beam; *l_mass_* is the length of the mass; *t* is time; p(y,τ) is the inertia force caused by vibration acceleration; and m(y,τ) is the external torque per unit length.

The surface strain of the beam can be represented by the following equation [25]:

(2) $$\varepsilon (y,t)=\frac{\partial^{2}z(y,t)}{\partial y^{2}}\frac{h}{2}$$

where ε(y,t) is the surface strain; *h* is the thickness of the beam.

According to Equations (1) and (2), ε(y,t) can be represented as

(3) $$\varepsilon (y,t)=\frac{h}{2}\frac{\partial^{2}}{\partial y^{2}}(\sum_{j=1}^{\infty }\frac{1}{\omega_{j}}Y_{j}(y)\int_{0}^{l+l_{mass}}Y_{j}(y)\times \int_{0}^{t}[p(y,\tau )-\frac{\partial }{\partial y}m(y,\tau )]\sin\omega_{j}(t-\tau )d\tau dy)$$

### 2.3. Geometry Parameters Optimization

The FBGs used in this work were made by UV exposure. A length of 5 mm was selected to ensure sensing accuracy and adequate resolution.

**Figure 3:**
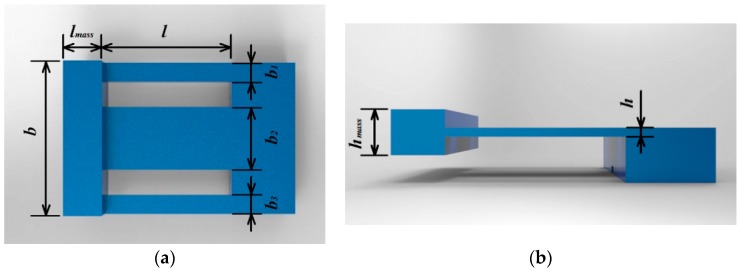
Illustration of simplified vibration gainer. (**a**) Top view; (**b**) Side view.

In this step, beam 2 was modeled as a constant-section beam to simplify the process of parameters optimization, as shown in Figure 3. According to the theory of vibration [24], the vibration amplitude of the beam increases with its decreasing thickness when mass is constant. Considering the strength of the beam and processing cost, thickness (*h*) and length (*l*) were set as 0.5 mm and 10 mm, respectively, to achieve miniaturization.

**Figure 4:**
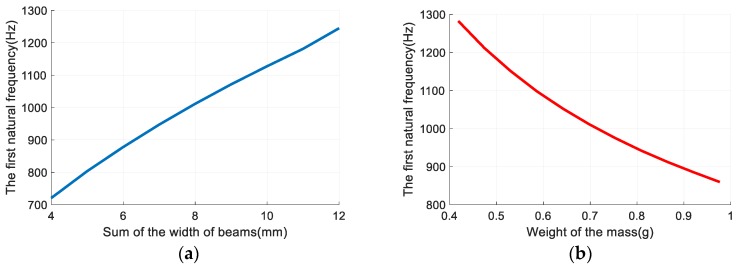
Optimized width of beams and weight of mass. (**a**) The first natural frequency changes with the sum of the width of beams when the mass is 0.7 g; (**b**) The first natural frequency changes with mass when the sum of the width of the beams is 8 mm.

Finite element analysis (FEA) was carried out to reveal changes of the natural frequency of the vibration gainer with changes in the sum of the width of beams (*b_sum_*) and the weight of the mass (*M_mass_*). The results are shown in Figure 4. It can be seen that the natural frequency increased with *b_sum_* and decreased with *M_mass_*. To ensure that the sensor can detect a wide range of vibrations, *b_sum_* was set as 8 mm and *M_mass_* was set as 0.7 g. The size of the mass was set as 3 mm × 2.5 mm × 12 mm (*l_mass_* × *h_mass_* × *b_mass_*). Under this condition, the first natural frequency of the sensor was 1011.5 Hz. To conveniently attach FBG 2, beam 2 should not be too narrow, so the width was set as 5 mm. Beam 1 and beam 3 help to improve the torsion resistance of the vibration gainer.

In the field of engineering, torsion resistance is typically characterized by the twist angle of unit length, *Φ*. To achieve symmetry, the width of beam 1 and 3 were set to be the same (1.5 mm). Then, the twist angle of the gainer can be obtained by the following equation:

(4) $$\Phi =\frac{3N}{G(b_{2}+2b_{1})h^{3}+\frac{3b_{mass}{}^{2}b_{1}h^{3}E}{4l^{2}}}$$

where *N* is the applied torque; *G* is the Shear Modulus; *b_sum_* is the width of vibration gainer; *b*_1_ is the width of beam 1; *b*_2_ is the width of beam 2; *h* is the thickness of beams; *E* is the Elastic Modulus; and *l* is the length of beams. If *N* = 0.01 Nm, the twist angle will be 0.266 rad/m. If the three beams are replaced by one beam with a width of *b_sum_*, the twist angle will be 0.407 rad/m. Thus, beam 1 and beam 3 increase the torsion rigidity by 34.6%.

**Figure 5:**
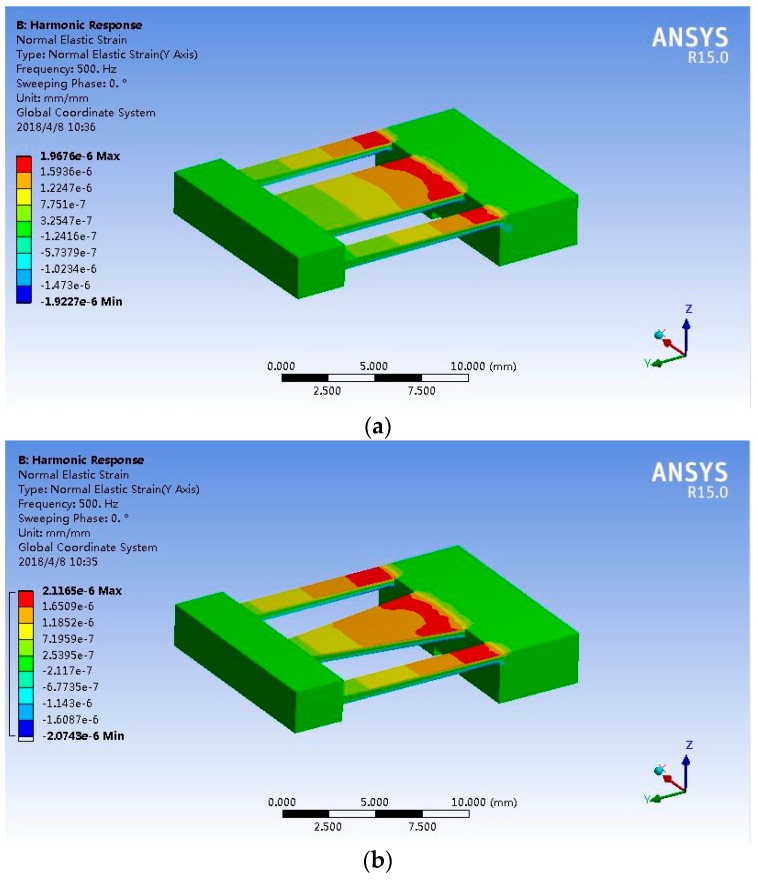
Strain distribution obtained by finite element analysis (FEA). (**a**) Constant-cross-section beam; (**b**) Optimized trapezoidal beam.

From the FEA result shown in Figure 5a, the root of beam 2 has the largest strain while sensing vibrations, therefore, one end of FBG 2 should be attached to the root to improve the sensing sensitivity, as shown in Figure 1a. However, the generated strain is non-uniform along the beam, which may widen the spectrum of FBG 2 or even split the spectrum into several peaks, reducing sensing accuracy. To reduce the gradient of the non-uniform strain, beam 2 was optimized as a trapezoid, as shown in Figure 5b. Considering the strength of the cantilever beams, the upper side of beam 2 (*b_2upper_*) was set as 2 mm. The strain gradient in the sensing area of the trapezoidal beam was 2.3 × 10^−2^ με/mm, which was a reduction of 78.0% compared to the beam with constant cross section. The first natural frequency of the vibration gainer changed to 956.56 Hz, which was slightly lower than that of the constant-section-beam vibration gainer (1011.5 Hz), and it is acceptable. Non-uniform strain can still widen the reflection spectrum of FBG 2. However, the center wavelength is used to demodulate the vibration signals so that the non-uniformly strain barely influences measurement results. All of the optimized geometry parameters and material properties of the stainless steel are summarized in Table 1.

## 3. Experiments and Analysis

**Figure 6:**
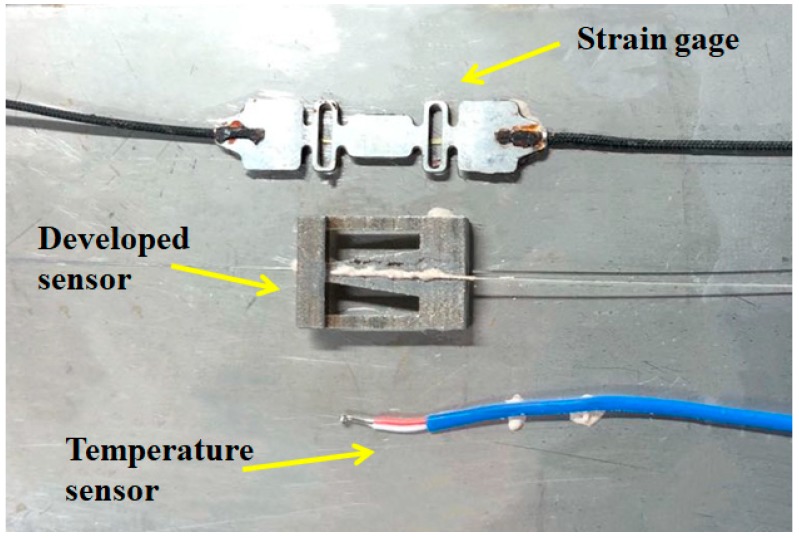
Schematic of the developed sensor.

Based on the designed geometry parameters, the sensor has been developed, as shown in Figure 6. It was attached to an object to measure strain, vibration, and temperature. The vibration gainer was made of stainless steel and the object selected to be measured was a 0.8 mm-thickness stainless steel plate. A SuperHawk 3120 strain gage and a temperature sensor were also attached to the object beside the newly designed sensor. Sensitivity of the strain gage was 1.401 pm/με and resolution of the temperature sensor was 0.1 °C. Since the temperature sensor was placed in close proximity to the newly designed sensor, the measured temperature values were the same. Therefore, the temperature sensor could be used to calibrate the newly designed sensor in temperature measurement. Similarly, strains measured by the strain gage were the same as those measured by the newly developed sensor. Therefore, the strain gage could be used to calibrate the newly designed sensor in strain measurement.

**Figure 7:**
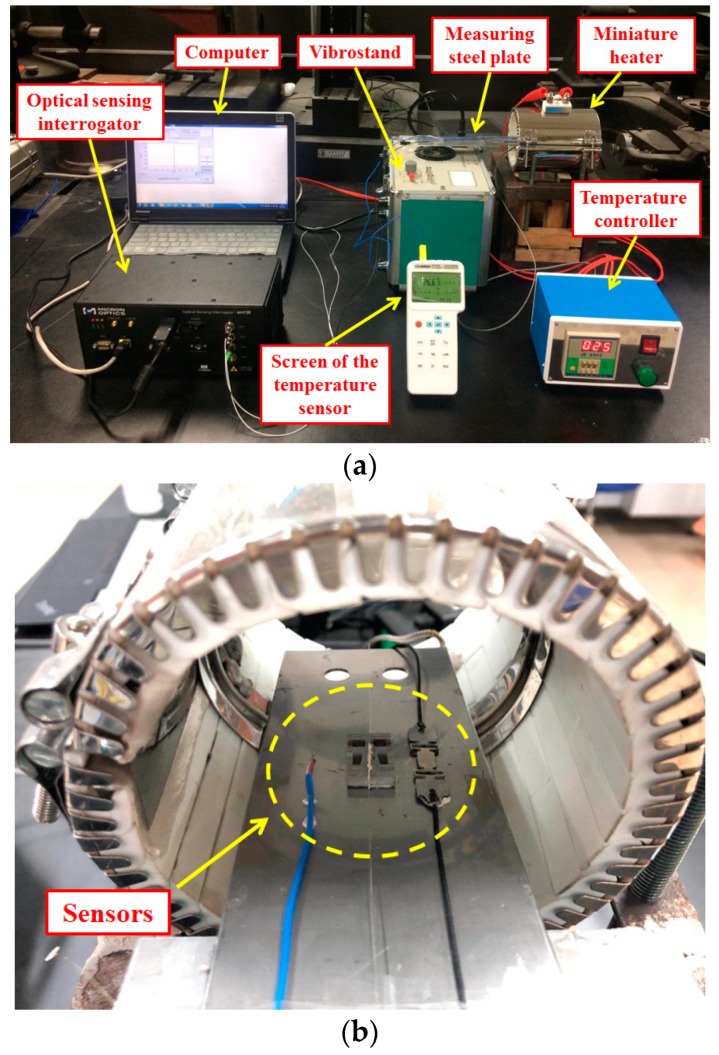

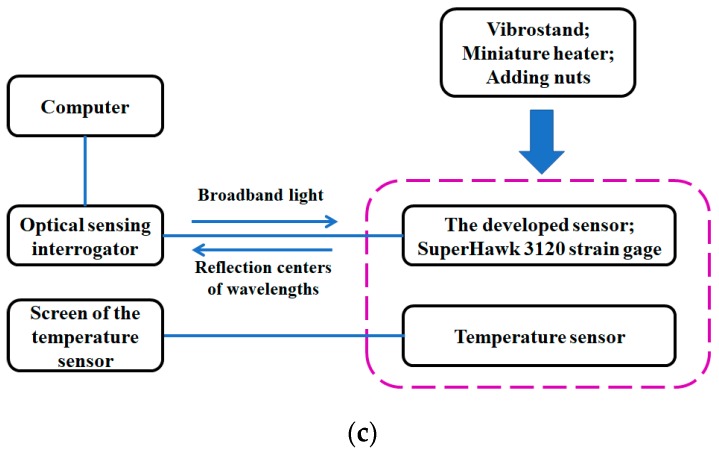
Experimental setup for testing the sensor. (**a**) Equipment used in experiment. (**b**) Sensors inside the miniature heater. (**c**) Block diagram of experimental procedure.

The experimental setup is shown in Figure 7a. A “SM130--700” dynamic optical sensing interrogator (1-kHz scanning frequency on 4 parallel channels simultaneously) was used to demodulate signals obtained by FBGs and strain gage. The vibration, strain, and temperature data were recorded and analyzed by the interrogator on a desktop computer. A vibrostand (SS-VSC-1) was used to generate vibrations. One end of the steel plate was attached to the vibrostand. Nuts were glued to the other end of the plate to generate surface strain. The sensors were attached onto the surface of the plate using a high-temperature-resistant glue (OMEGABOND “600”). Part of the plate and sensors were placed inside a miniature heater, as shown in Figure 7b and a temperature controller was used to control the temperature inside the miniature heater.

A schematic block diagram of the setup is provided in Figure 7c. The broadband light provided by the optical sensing interrogator was transmitted to the newly developed sensor and the SuperHawk 3120 strain gage via optical fibers. Then, the reflection center wavelengths were transmitted back to the interrogator via the same optical fibers and the output data were recorded and analyzed on the computer. Changes in temperature, vibration, and strain were achieved by using a miniature heater, vibrostand, and adding nuts, respectively.

Individual and simultaneous sensing experiments were carried out to measure strain, temperature, and vibration. The individual experiments were used to calibrate the performance of the newly designed sensor, since individual measurements are simpler and more accurate than simultaneous measurements. After obtaining equations for describing the strain, temperature, and vibration using the individual sensing experiments, simultaneous sensing was used to verify the equations. The data were compared to assess the performance of the newly developed sensor in simultaneous measurements.

### 3.1. Individual Measurement

In this section, we present results of the individual sensing experiment and use them to determine performance of the sensor for strain, temperature, and vibration measurements. As mentioned above, sensors were attached to a steel plate. The rest of the equipment was connected as shown in Figure 7b,c.

#### 3.1.1. Strain Measurement

To calibrate the strain sensing performance of the sensor, the vibrostand was turned off and temperature was held at 30 °C. Strain was applied to the steel plate by adding nuts at the free end of the plate. At the beginning of the experiment, four nuts were glued to the free end of the plate. Meanwhile, shifts in the center wavelengths of FBG 1 and the strain gage were detected and recorded. Four nuts were added at a time until all 24 nuts were attached to the free end of the plate and six group sets of experimental data were obtained. The traditional strain gage was placed in close proximity to the newly developed sensor. Therefore, we assume that the FBG 1 measured the same strain as the strain gage, calculated as follows:

(5) $$\varepsilon =\frac{\Delta \lambda_{S}}{S_{g}}$$

where *ε* is strain; $\Delta \lambda_{S}$ is the shift of center wavelength of the strain gage; *S_g_* is the sensitivity of the strain gage.

As the shift of center wavelength of FBG 1 exhibits a linear relationship with the applied strain, a regression equation can be used to describe this relationship. The regression equation obtained using least squares method to fit the shift of FBG 1 and the strain value is

(6) $$\Delta \lambda_{\varepsilon 1}=1.878\varepsilon -10.486$$

where *ε* is strain; Δ*λ_ε_*_1_ is the change of center wavelength of FBG 1.

**Figure 8:**
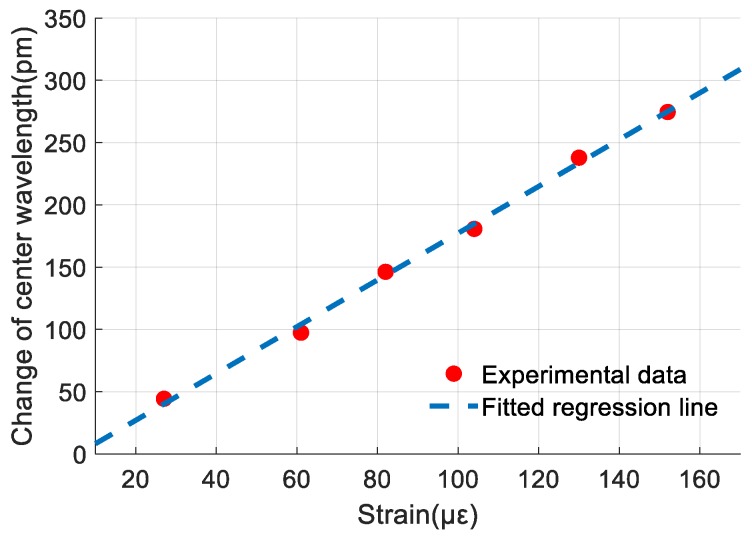
The relationship between reflection center of Fiber Bragg grating (FBG) 1 and strain.

Equation (6) shows that the sensitivity of FBG 1 for strain measurement is 1.878 pm/με. Figure 8 presents the experimental data of FBG 1 and the fitted regression line. The maximum strain measured in this work was 152 με.

The experimental data and the regression line can be used to obtain the nonlinearity error of FBG 1 for strain measurement. The nonlinearity error (*δ_Nonlinearity_*) is defined using the following equation [26]:

(7) $$\delta_{Nonlinearity}=\frac{\delta_{Max}}{\Delta \lambda_{Max}}\cdot 100\%$$

where *δ_Max_* is the absolute value of the maximum differences between the regression line and the measurement data; Δ*λ_Max_* is the maximum measured value in the measurement range. In this work, the nonlinearity error of FBG 1 for strain measurement is 2.42% F.S.

**Figure 9:**
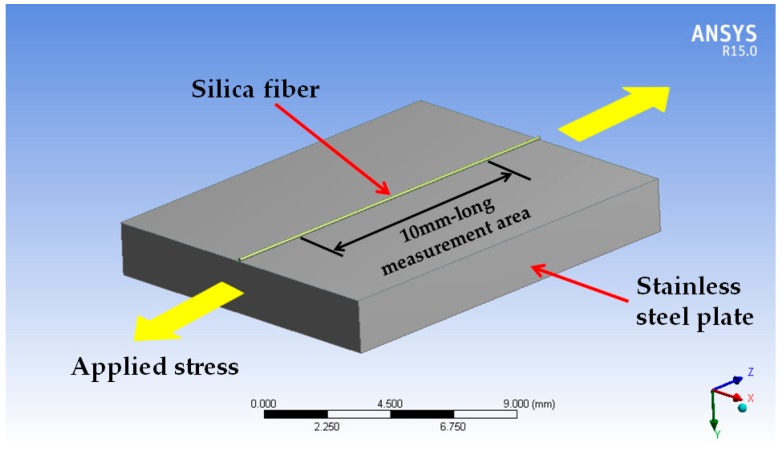
Three-dimensional illustration of the stainless-steel plate with a 15 mm-long silica fiber attached to the surface.

The FBG has an elastic modulus of 72 GPa and a diameter of 0.125 mm, which is too small to disturb the strain distribution of the measured object, thus, the measured strain is the real surface strain of the object. To verify this conclusion, FEA was carried out. Briefly, the measured object was set as a stainless-steel plate with dimensions 15 mm × 12 mm × 2 mm. A 15-mm-long silica fiber was glued onto the surface of the steel, as shown in Figure 9.

**Figure 10:**
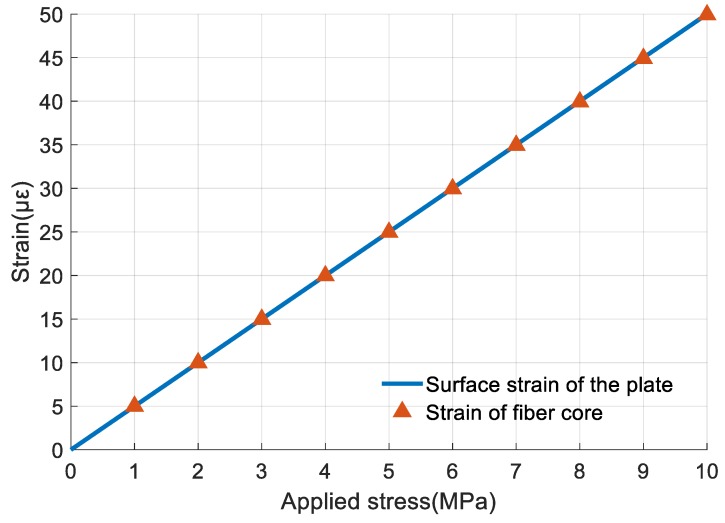
Comparison of surface strain of steel plate and strain of fiber core.

By applying different values of stress to the plate, the strain within the 10-mm-long measurement area in the middle of the fiber was simulated. Compared to surface strain on the plate without the fiber, the maximum deviation in strain is shown in Figure 10. From the figure, strain of fiber core fits very well with the surface strain of the plate. The maximum deviation appears at 8 MPa and is 0.188% which is much smaller than the nonlinearity error of the strain measurement. Thus, the deviation between strain at the FBG core and on the surface of the measured object can be ignored. Strain measured by FBG 1 represents the real surface strain of the object.

#### 3.1.2. Temperature Measurement

To calibrate the temperature sensing performance of the sensor, the vibrostand was turned off and the stainless-steel plate was strain-free. The miniature heater was turned on and the temperature was increased from 50 °C to 210 °C at 10 °C increments. The center wavelengths of the two FBGs and temperature measured by temperature sensor were recorded throughout the experiment. 

Changes in the center wavelength of the FBGs are linearly related to temperature, therefore, regression equations can be used to describe their relationships. The least squares method was used to analyze the recorded data and regression equations were obtained, as follows:

(8) $$\{\begin{array}{l} \Delta \lambda_{T1}=19691T-761.35\\ \Delta \lambda_{T2}=29.324T-1185.3 \end{array}$$

where *T* is temperature; $\Delta \lambda_{T1}$ and $\Delta \lambda_{T2}$ are the changes of center wavelengths of FBG 1 and FBG 2.

Equation (8) shows sensitivities of FBG 1 and FBG 2 for temperature measurement of 19.691 pm/°C and 29.324 pm/°C, respectively. These sensitivities are different, mainly due to the differences in the thermal expansion coefficients. The sensitivity of FBG 2 for temperature measurement is higher than that of bare FBGs. This is also caused by thermal expansion of beam 2.

**Figure 11:**
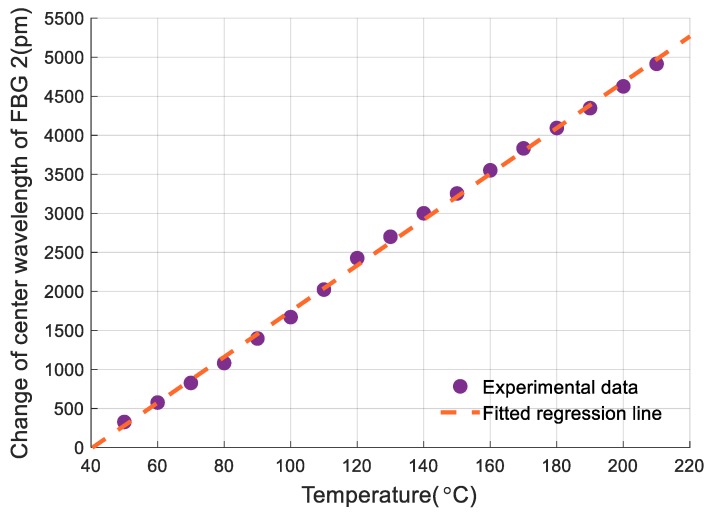
Relationship between temperature and the reflection center of Fiber Bragg grating (FBG) 2.

As mentioned in Section 2.1, FBG 2 is used for sensing temperature. Figure 11 shows the experimental data of FBG 2 and its fitted regression line.

Based on the experimental data and the definition of nonlinearity error presented in Section 3.1.1, the nonlinearity error of FBG 2 for temperature measurement is 1.88% F.S., and appears at 120 °C.

When FBG 1 is affected by both strain and temperature changes, the shift of its center wavelength can be represented as

(9) $$\Delta \lambda_{1}=\Delta \lambda_{\varepsilon 1}+\Delta \lambda_{T1}$$

where $\Delta \lambda_{\varepsilon 1}$ is the shift of center wavelength caused by strain, $\Delta \lambda_{T1}$ is the shift of center wavelength caused by the change of temperature. Combining Equations (8) and (9), the modified strain measurement equation can be obtained as

(10) $$\Delta \lambda_{\varepsilon }=\Delta \lambda_{1}-19.691T+761.35$$

#### 3.1.3. Vibration Measurement

While calibrating vibration sensing performance of the sensor, the steel plate was kept strain-free and the temperature was held constant at 30 °C. The vibrostand was turned on and the applied vibration acceleration was gradually increased from 10 m/s^2^ to 55 m/s^2^. The shift of center wavelength of FBG 2 was detected and recorded throughout the experiment and analyzed by FFT to obtain the vibration signal.

First, the average of the measurement data was calculated and subtracted from the data. Then, the rest of the data was analyzed by FFT to obtain the frequency spectrum. Values that did not occur at 0 Hz were linearly related to the amplitude of vibration, therefore, a regression equation was used to describe this relationship. Using least squares method, the regression equation can be obtained as

(11) $$\Delta \lambda_{a2}=0.769a+2.935$$

where Δ*λ_a_*_2_ is the amplitude of center wavelength of FBG 2; *a* is vibration acceleration.

**Figure 12:**
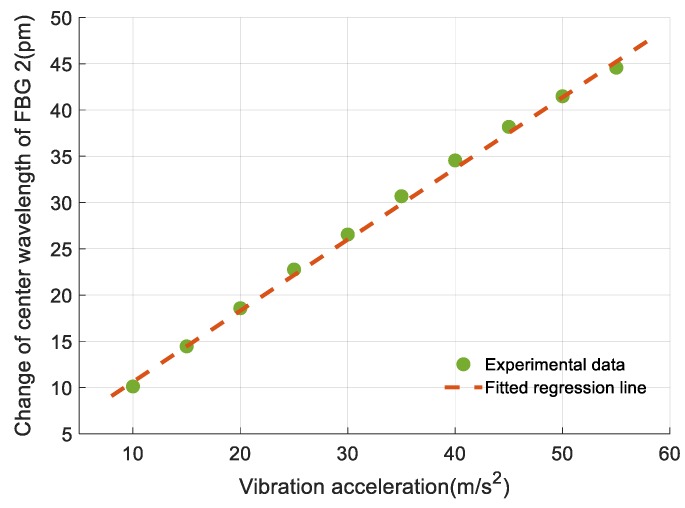
Relationship between the amplitude of the frequency spectrum of Fiber Bragg grating (FBG) 2 and vibration acceleration.

Equation (11) shows that the sensitivity of FBG 2 for vibration measurement is 0.769 pm/m·s^−2^. Figure 12 shows the experimental data of FBG 2 and its fitted regression line. The nonlinearity error of FBG 2 for vibration measurement is obtained according to Section 3.1.1 and experimental data and it is 1.83% F.S. at 40 m/s^2^.

### 3.2. Simultaneous Measurement

In this section, simultaneous sensing experiments are presented and performance of the sensor for strain, temperature, and vibration measurements is assessed. The equipment was connected as shown in Figure 7b,c. The set temperature, vibration acceleration, and strain are shown in Table 2.

Analysis of the data measured by FBG 1 and FBG 2 was divided into two steps. First, data measured by FBG 2 was analyzed to obtain the temperature and vibration. Second, data measured by FBG 1 was modified using Equation (10) to obtain the strain. According to Section 2.1, the sensing system and analyzing system can work simultaneously, so the signal separation method can be used in dynamic vibration detection.

#### 3.2.1. Separated of Temperature and Vibration

As mentioned in Section 2.1, a one-second-long signal was intercepted from the signal sensed by FBG 2. Then, the average value was subtracted from the original data. The average value represented temperature. The rest of the data was analyzed by FFT to obtain the frequency spectrum. Values at non-zero frequencies of the frequency spectrum represented vibration.

**Figure 13:**
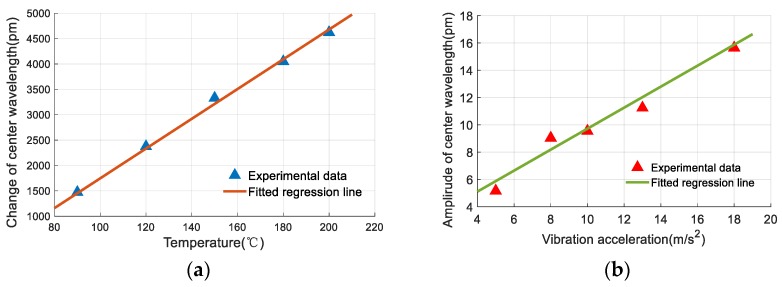
Separated temperature and vibration by the interrogation method. (**a**) Temperature; (**b**) Vibration.

The average values obtained to represent temperature were compared with the fitted regression line obtained in Section 3.1.2, as shown in Figure 13a. The nonlinearity error, as mentioned in Section 3.1.1, was then used to measure the error between the average values and the regression line. The result shows a maximum error of 1.89% F.S. at 150 °C, which is similar to the nonlinearity error obtained while measuring temperature individually, presented in Section 3.1.2. Similarly, values at non-zero frequencies of the frequency spectrum, which represented vibration, were compared with the fitted regression line obtained in Section 3.1.3, as shown in Figure 13b. The nonlinearity error was used to measure the error between the experimental data and the regression line. The results show that the maximum error appears at 8 m/s^2^ and its value is 2.23% F.S., which is slightly higher than the nonlinearity error mentioned in Section 3.1.3. This is caused by thermal expansion of beam 2.

As errors in simultaneous measurements of temperature and vibration are almost the same as those errors in individual measurements, results of the simultaneous sensing experiment match well with Equations (8) and (11). This shows that Equations (8) and (11) can be used to describe temperature and vibration sensing performance when the developed sensor is used for simultaneous measurements.

#### 3.2.2. Modified of Strain Measurement

**Figure 14:**
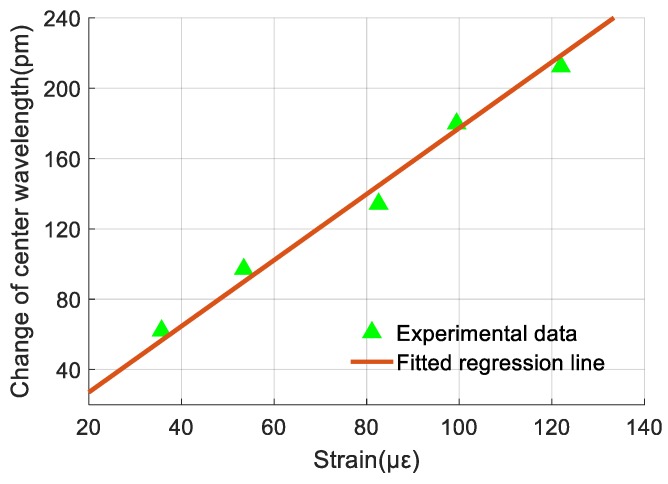
Modified strain by the interrogation method.

According to Equation (10), the measured temperature was used to modify the strain which sensed by FBG 1. The modified value $\Delta \lambda_{\varepsilon }$ was then compared with the fitted regression line obtained in Section 3.1.1, as shown in Figure 14. The nonlinearity error defined by Equation (7) was used to measure error between the modified strain and the regression line. The maximum error appears at 82.6 με with a value of 4.23% F.S., which is higher than the nonlinearity error in individual measurements, as presented in Section 3.1.1. The reason for this increase is that when temperature and strain are measured simultaneously, the nonlinearity error of the temperature measurement will affect the strain measurement. The error transfer equation can be expressed as

(12) $$\delta =\sqrt{\delta_{\varepsilon }{}^{2}+\delta_{T}{}^{2}}$$

where δ is the error of the modified strain measured by FBG 1; $\delta_{\varepsilon }$ is the nonlinearity error of the individually measured strain; $\delta_{T}$ is the nonlinearity error of the individually measured temperature.

Since the nonlinearity errors of temperature and strain were 1.89% F.S. and 2.43% F.S. respectively, according to Section 3.2 and Section 3.1.1, the maximum error of strain in simultaneous measurements should be higher than 3.08% F.S. according to Equation (12). The error of 4.23% F.S. fits this requirement.

As the maximum error of strain in the simultaneous measurements is similar to the value of individual measurements, results of the simultaneous sensing experiment match well with Equation (10). This shows that Equation (10) can be used to describe strain sensing performance when the developed sensor is used for simultaneous measurements.

## 4. Conclusions

A combined strain-vibration-temperature sensor has been developed, comprising FBG 1, FBG 2, and a vibration gainer. Geometry parameters of the sensor were optimized in terms of the results of FEA, processing cost, and strength of the structure. Experimental measurements of strain, vibration, and temperature were carried out, and an interrogation method was developed to eliminate interactions between strain, vibration, and temperature. The results show that the newly developed sensor can sense strain, vibration, and temperature, both individually and simultaneously.

The performance of the sensor for individually sensing strain was obtained using Equation (6). The sensitivity and nonlinearity error in the working range of 0–152 με were 1.878 pm/με and 2.43% F.S., respectively. The temperature sensing performance can be represented as $\Delta \lambda_{T2}$ in Equation (8). The sensitivity and nonlinearity error in the working range of 50–210 °C were 29.324 pm/°C and 1.88% F.S., respectively. Finally, the performance of vibration sensing was determined by Equation (11). The sensitivity and nonlinearity error in the working range of 10–55 m/s^2^ were 0.769 pm/m·s^−2^ and 1.83% F.S., respectively.

For simultaneous sensing, the proposed interrogation method mentioned in Section 2.1 was used to eliminate interactions between strain, vibration, and temperature. After the process, data for temperature and vibration matched well with the values obtained using Equations (8) and (11). This shows that Equations (8) and (11) can be used to describe temperature and vibration sensing performance of the newly developed sensor for simultaneous measurements. The nonlinearity errors were 1.89% F.S. and 2.23% F.S., respectively. Similarly, after using interrogation method, data representing strain matched the values obtained using Equation (10). This shows that Equation (10) can be used to describe strain sensing performance of the newly developed sensor for simultaneous measurements. The nonlinearity error was 4.23%, which was higher than that of individual strain sensing but this can be explained by the Equation (12), the error transfer equation.

## Figures and Tables

**Table 1:** Optimized geometry parameters of sensor.

Geometry Parameters	Value	Geometry Properties	Value	Material Properties	Value
*l* (m)	10 × 10^−3^	*b*_2*upper*_ (m)	2 × 10^−3^	*E* (GPa)	193
*h* (m)	0.5 × 10^−3^	*b*_3_ (m)	1.5 × 10^−3^	*G* (GPa)	73.7
*b_sum_* (m)	8 × 10^−3^	*l_mass_* (m)	3 × 10^−3^	*ρ* (kg∙m^−3^)	7750
*b*_1_ (m)	1.5 × 10^−3^	*h_mass_* (m)	2.5 × 10^−3^	*υ*	0.31
*b*_2_ (m)	5 × 10^−3^	*b_mass_* (m)	12 × 10^−3^		

**Table 2:** Set values of vibration acceleration, strain, and temperature.

Serial Number	Temperature (°C)	Vibration Acceleration (m/s^2^)	Strain (με)
1	90	5	35.7
2	120	10	53.5
3	150	13	82.6
4	180	8	99.4
5	200	18	121.9
